# The impact of socioeconomic status and sleep quality on the prevalence of multimorbidity in older adults

**DOI:** 10.3389/fpubh.2022.959700

**Published:** 2022-09-26

**Authors:** Benli Xue, Yaqing Xue, Fang Dong, Xiao Zheng, Lei Shi, Shujuan Xiao, Jiachi Zhang, Weiyan Ou, Qi Wang, Chichen Zhang

**Affiliations:** ^1^School of Health Management, Southern Medical University, Guangzhou, China; ^2^School of Public Health, Southern Medical University, Guangzhou, China; ^3^Shenzhen People's Hospital (The Second Clinical Medical College, Jinan University, The First Affiliated Hospital, Southern University of Science and Technology), Shenzhen, China; ^4^Department of Health Management, Nanfang Hospital, Southern Medical University, Guangzhou, China; ^5^Institute of Health Management, Southern Medical University, Guangzhou, China

**Keywords:** older adults, socioeconomic status, sleep quality, multimorbidity, prevalence

## Abstract

**Introduction:**

Multimorbidity has become a global public health concern that can cause serious damage to the health status of older adults. This study aimed to investigate the impact of socioeconomic status (SES) and sleep quality on the prevalence of multimorbidity in older adults, thus providing a reference for reducing the risk of the prevalence of multimorbidity and improving the health of older adults.

**Methods:**

A multi-stage random sampling method was used to conduct a questionnaire survey on 3,250 older adults aged 60 years and above in Shanxi Province, China. The chi-square test and multiple logistic regression models were used to analyze the association of SES and sleep quality with the prevalence of multimorbidity of older adults.

**Results:**

The prevalence of multimorbidity was 30.31% in older adults aged 60 years and above in Shanxi Province, China. After adjusting for confounders, very low SES (OR = 1.440, 95% CI: 1.083–1.913) and poor sleep quality (OR = 2.445, 95% CI: 2.043–2.927) were associated with the prevalence of multimorbidity. Older adults with low SES and poor sleep quality had the highest risk of the prevalence of multimorbidity (OR = 3.139, 95% CI: 2.288–4.307).

**Conclusions:**

SES and sleep quality are associated with the prevalence of multimorbidity in older adults, and older adults with lower SES and poorer sleep quality are at higher risk for the prevalence of multimorbidity.

## Introduction

Aging is one of the most prominent features in recent population dynamics around the world. Owing to the declines in both fertility and mortality, the proportion of older adults in the population has increased substantially both in developed and developing countries (1). China has experienced rapid population aging in the past decades (2). According to the seventh national census, the population aged 60 and above has exceeded 264 million, accounting for 18.70% of the total population of China (3). The population aging brings a series of socioeconomical challenges to the current healthcare system in China (4).

Multimorbidity is defined as the condition of co-existence of two or more chronic conditions in a person (5). It is becoming more and more common in older adults. A systematic review (41 articles were included) indicated that the prevalence of multimorbidity in older adults ranges from 55.0 to 98.0% (6). Multimorbidity can lead to adverse health consequences for older adults. A study revealed the risk of death was 1.73 and 2.72 times for older adults with 2+ and 3+ morbidities, respectively, compared to those without multimorbidity (7). Multimorbidity is influenced by several factors. Studies found a strong association between multimorbidity and age (8, 9). Compared with men, women are at higher risk of multimorbidity (10). In addition, several factors, such as low education level, smoking, alcohol consumption, obesity, high waist circumference, and family history of chronic conditions, are associated with multimorbidity (10–12). Focusing on multimorbidity of older adults is of great significance to protect their health and cope with population aging.

Among various health-related factors, SES holds an important position. In terms of relationships between SES, health, and social causality, there is a common view among scholars that the lower the individual's SES, the worse the health status (13). Scholars found that SES is one of the strongest and most consistent predictors of morbidity and mortality (14–16). This finding applies to all diseases with few exceptions, and SES continues throughout the entire life span (14). However, is the effect of SES on multimorbidity in older adults the same? Based on this, we hypothesize that there is a significant association between SES and the prevalence of multimorbidity in older adults.

Sleep, as a life behavior occupying a significant portion of our lives, plays an important role in maintaining human health (17). But sleep disorder has become one of the major health issues of older adults (18, 19). Epidemiological studies have shown an association between poor sleep quality and perennial chronic diseases in older adults, such as type 2 diabetes, hypertension, coronary heart disease, and chronic obstructive pulmonary disease (20, 21). Sleep quality is also associated with psychological distress, and older adults who have poor sleep quality are more likely to experience various mental health problems (22). Therefore, we hypothesize that poor sleep quality is related to the prevalence of multimorbidity in older adults. Different SES will greatly affect people's behavior and lifestyle (23). Several studies have found that people with low SES has worse sleep than those with high SES (24–26). While improving sleep quality is an effective way to protect health, whether this is effective for older adults with any SES and the effect of different sleep qualities on the prevalence of multimorbidity in older adults with different SES are unclear. Therefore, this study assumed that different combinations of SES and sleep quality have different effects on the prevalence of multimorbidity in older adults to analyze the combined association of SES and sleep quality.

The purpose of this study was to investigate the impact of SES and sleep quality on the prevalence of multimorbidity in older adults. In the context of population aging, the health of older adults is receiving increasing attention. It is believed that the results of this study would contribute to reducing the risk of the prevalence of multimorbidity and improving their health.

## Materials and methods

### Sample and participants

A cross-sectional study based on a structured questionnaire was conducted in Shanxi Province from June to August 2019, and a multi-stage random sampling method was used to select participants. In the first stage, all 11 prefecture-level cities in Shanxi Province were included as sampling units, and the districts (counties and county-level cities) of each prefecture-level city were numbered according to the order published on the government website. In the second stage, two districts (counties and county-level cities) were randomly selected from each prefecture-level city using the random number table method. In the third stage, two communities (administrative villages) were selected from each district (county and county-level city) using the same method. In the fourth stage, considering the different population sizes of each community (administrative village), one or two residential communities (natural villages) were selected from each community (administrative village), from which older adults were randomly selected as study subjects.

The inclusion criteria were as follows: permanent residents of Shanxi Province aged 60 years and above, without serious cognitive impairment, with no communication impairment, and voluntarily participation in the survey. A total of 3,266 questionnaires were distributed, and 3,250 valid questionnaires were recovered, with an effective recovery rate of 99.51%.

All the participants were informed of the purpose and procedure of the investigation, after which they signed consent. After providing consent, the participants were invited by trained investigators to respond to questionnaires. All questionnaires were anonymous. The study procedures were approved by the University Ethics Committee.

### Measures

#### Socioeconomic status

Factors such as education level, occupation, income, wealth, and deprivation are often used to reflect SES (2). This study selected educational level, occupation before retirement, and personal monthly income to measure the SES of older adults with reference to a previous study (23). Educational level is divided into the following four categories: no formal education, primary education, secondary education, and higher education. Occupation before retirement is divided into the following five groups: unemployed, peasant, worker, professional technicians, and enterprise/institution personnel. Personal monthly income (RMB) is divided into three levels: less than 1,000, 1,000–2,999, and 3,000 and above. Principal component analysis (PCA) was used to construct the SES index to comprehensively evaluate the SES of the older adults. Quartiles were used to classify the SES index into four categories: very high, high, low, and very low.

#### Sleep quality

The Pittsburgh Sleep Quality Index (PSQI) was used to measure sleep quality of older adults (27). It contains seven component scores: subjective sleep quality, sleep latency, sleep duration, habitual sleep efficiency, sleep disturbances, use of sleep medications, and daytime dysfunction. The seven component scores were added to yield a global PSQI score, and the range of the global score was 0–21. The higher the score, the worse the sleep quality. In this study, the global PSQI scores of <5 and ≥5 were defined as “good sleep quality” and “poor sleep quality”, respectively (28). Cronbach's α was 0.758 for the PSQI in this study.

#### Multimorbidity

A total of 26 chronic diseases were included in the questionnaire in this study (e.g., hypertension, diabetes, coronary heart disease, atherosclerosis, chronic obstructive pulmonary disease, chronic nephritis, stroke, osteoporosis). The number of chronic diseases for older adults was self-reported and verified with the diagnostic evidence of medical records or the prescriptions from doctors (29). Multimorbidity was established when older adults suffer from two or more chronic diseases.

#### Covariates

Sociodemographic and lifestyle variables of older adults were recorded through the questionnaire. Sociodemographic variables included gender (male or female), age (60-, 70-, or 80- years), marital status (married, unmarried or other), empty nest status (not-empty nest, empty nest, or no children), family history of diseases (no or yes), and BMI categories (underweight [<18.5 kg/m^2^], normal weight [18.5- kg/m^2^], overweight [24- kg/m^2^], or obesity [≥28 kg/m^2^]). Lifestyle variables included physical activity level (low, middle, or high), drinking status (not-drinking, past-drinking, or now-drinking), and smoking status (not-smoking, past-smoking, or now-smoking). Physical activity level was measured using the Chinese version of the International Physical Activity Questionnaire, long form (IPAQ-LC), and the IPAQ-LC has demonstrated adequate reliability and showed sufficient evidence of validity (30). The results were classified into low, middle, and high categories according to the criteria accordingly (31).

### Statistical analysis

Continuous variables were presented as mean ± SE, and categorical variables as frequency (%). PCA was used to construct the SES index. The chi-square test was used to compare the prevalence of multimorbidity among older adults with different sociodemographic variables, lifestyle variables, sleep quality, and SES. The multiple logistic regression models were used to analyze the association of SES and sleep quality with the prevalence of multimorbidity, and the association of different combinations of SES and sleep quality with the prevalence of multimorbidity in older adults. SPSS 22.0 (IBM, New York, USA) was used for all the statistical analyses in this study. All tests were two-tailed, and *P* < 0.05 was considered statistically significant.

## Results

### Basic characteristics of older adults

Among the 3,250 older adults, 1,515 (46.62%) were male and 1,735 (53.38%) were female. The largest number [1,769 (54.43%)] of older adults aged 60–70 years. A total of 985 (30.31%) participants suffered from multimorbidity, and 1,587 (48.83%) participants were recognized as having poor sleep quality. The distribution of the sociodemographic variables of older adults is shown in Table 1.

**Table 1:** Multimorbidity with sociodemographic, lifestyles, and sleep quality in older adults.

		**Types of chronic diseases [*N* (%)]**				
**Factors**	**N**	**None**	**1**	**≥2 (multimorbidity)**	**χ^2^**	***P***
**Gender**					6.890	0.032
Male	1,515	652 (43.04)	438 (28.91)	425 (28.05)		
Female	1,735	697 (40.17)	478 (27.55)	560 (32.28)		
**Age**					127.342	<0.001
60-	1,769	872 (49.29)	468 (26.46)	429 (24.25)		
70-	1,164	386 (33.16)	372 (31.96)	406 (34.88)		
80-	317	91 (28.71)	76 (23.97)	150 (47.32)		
**Marital status**					11.459	0.003
Married	2,842	1,061 (42.75)	705 (28.40)	716 (28.85)		
Unmarried or others	768	288 (37.50)	211 (27.47)	269 (35.03)		
**Empty nest status**					42.610	<0.001
Not-empty nest	1,599	703 (43.96)	424 (26.52)	472 (29.52)		
Empty nest	1,581	595 (37.63)	483 (30.55)	503 (31.82)		
No children	70	51 (72.86)	9 (12.86)	10 (14.28)		
**Family history of diseases**					88.183	<0.001
No	3,084	1,334 (43.26)	860 (27.89)	890 (28.86)		
Yes	166	15 (9.04)	56 (33.73)	95 (57.23)		
**BMI categories**					35.434	<0.001
Underweight	452	202 (44.69)	111 (24.56)	139 (30.75)		
Normal weight	1,719	771 (44.85)	451 (26.24)	497 (28.91)		
Overweight	843	301 (35.71)	280 (33.21)	262 (31.08)		
Obesity	236	75 (31.78)	74 (31.36)	87 (36.86)		
**Physical activity level**					20.474	<0.001
Low	723	317 (43.85)	162 (22.41)	244 (33.74)		
Middle	1,321	527 (39.89)	382 (28.92)	412 (31.19)		
High	1,206	505 (41.87)	372 (20.85)	329 (27.78)		
**Drinking status**					49.057	<0.001
Not-drinking	2,389	1,025 (42.90)	650 (27.21)	714 (29.89)		
Past-drinking	279	62 (22.22)	102 (36.56)	115 (41.22)		
Now-drinking	582	262 (45.02)	164 (28.18)	156 (26.80)		
**Smoking status**					60.782	<0.001
Not-smoking	2,264	987 (43.60)	601 (26.55)	676 (29.85)		
Past- smoking	357	81 (22.68)	138 (38.66)	138 (38.66)		
Now-smoking	629	281 (44.67)	177 (28.14)	171 (27.19)		
**Sleep quality**					150.266	<0.001
Good	1,663	854 (51.35)	431 (25.92)	378 (22.73)		
Poor	1,587	495 (31.19)	485 (30.56)	607 (38.25)		
**Total**		1,349 (41.51)	916 (28.18)	985 (30.31)		

### Multimorbidity with different characteristics

The results showed that there were significant differences in gender, age, marital status, empty nest status, family history of diseases, BMI categories, physical activity level, drinking status, smoking status, and sleep quality in different types of chronic diseases (*P* < 0.05). The factors female, age 80 years and above, unmarried or others, empty nest, have a family history of diseases, obesity, low physical activity level, past-drinking, past-smoking, and poor sleep quality had a higher prevalence of multimorbidity (Table 1).

### SES of older adults

Among the 3,250 older adults, 577 (17.75%) had no formal education, while 1,062 (32.68%), 1,324 (40.74%), and 287 (8.83%) had primary, secondary, and higher education, respectively. In terms of occupation before retirement, the largest number [1,466 (45.11%)] of older adults were peasants, while the smallest number [221 (6.80%)] were unemployed. Of all older adults, 1,691 (52.03%) had a personal monthly income <1,000, followed by 808 (24.86%) with 3,000 and above and 751 (23.11%) with 1,000-2,999. PCA was used to construct the SES index. The results showed that the KMO coefficient was 0.680, and Bartlett's test showed *P* < 0.001. Based on the principle that the eigenvalue is more than 1 or the cumulative contribution rate is more than 80%, the principal component was determined and the SES index was calculated. Then, the SES index was divided into four categories based on quartiles: very high, high, low, and very low. The number of older adults in each category was 873 (26.86%), 753 (23.17%), 1,063 (32.71%), and 561 (17.26%), respectively (Table 2).

**Table 2:** Multimorbidity with SES in older adults.

		**Types of chronic diseases [*****N*** **(%)]**				
**Factors**	**N**	**None**	**1**	**≥2 (multimorbidity)**	**χ^2^**	***P***
**Educational level**					67.211	<0.001
No formal education	577	198 (34.32)	162 (28.08)	217 (37.60)		
Primary education	1,062	389 (36.63)	329 (30.98)	344 (32.39)		
Secondary education	1,324	596 (45.02)	364 (27.49)	364 (27.49)		
Higher education	287	166 (57.84)	61 (21.25)	60 (20.91)		
**Occupation before retirement**					15.608	0.048
Unemployed	221	98 (44.34)	59 (26.70)	64 (28.96)		
Peasant	1,466	560 (38.20)	428 (29.20)	478 (32.60)		
Worker	538	237 (44.05)	155 (28.81)	146 (27.14)		
Professional technicians	272	124 (45.59)	64 (23.53)	84 (30.88)		
Enterprise/institution personnel	753	330 (43.82)	210 (27.89)	213 (28.29)		
**Personal monthly income (RMB)**					20.963	<0.001
<1,000	1,691	654 (38.68)	476 (28.15)	561 (33.17)		
1,000–2,999	751	353 (47.00)	211 (28.10)	187 (24.90)		
≥3,000	808	342 (42.33)	229 (28.34)	237 (29.33)		
**SES**					30.696	<0.001
Very high	873	389 (44.56)	237 (27.15)	247 (28.29)		
High	753	349 (46.35)	203 (26.96)	201 (26.69)		
Low	1,063	419 (39.42)	318 (29.92)	326 (30.66)		
Very low	561	192 (34.22)	158 (28.16)	211 (37.62)		
**Total**		1,349 (41.51)	916 (28.18)	985 (30.31)		

### Multimorbidity with different SES

The results showed that there were significant differences in educational level, occupation before retirement, personal monthly income, and SES in different types of chronic diseases (*P* < 0.05). The prevalence of multimorbidity was higher among older adults for the factors no formal education, peasant, personal monthly income <1,000, and very low SES (Table 2).

### The association of SES and sleep quality with multimorbidity

The association of SES and sleep quality with the prevalence of multimorbidity was analyzed using the multiple logistic regression model. After adjusting for confounders, compared with very high SES, the risk of the prevalence of multimorbidity of high SES, low SES, and very low SES was 0.920 (95% CI: 0.713–1.188), 1.195 (95% CI: 0.943–1.515), and 1.440 (95% CI: 1.083–1.913), respectively. Compared with good sleep quality, the risk of the prevalence of multimorbidity was 2.445 (95% CI: 2.043–2.927) for poor sleep quality (Table 3).

**Table 3:** Association of SES and sleep quality with multimorbidity in older adults.

		**Types of chronic diseases**							
**Factors**	**N**	**0 vs. 1**				**0 vs**. ≥**2 (multimorbidity)**			
		**UOR (95% CI)**	***P***	**AOR (95% CI)**	***P***	**UOR (95% CI)**	***P***	**AOR (95% CI)**	***P***
**SES**									
Very high	873	Reference	–	Reference	–	Reference	–	Reference	–
High	753	0.927 (0.730–1.177)	0.534	0.982 (0.765–1.260)	0.884	0.866 (0.681–1.102)	0.242	0.920 (0.713–1.188)	0.523
Low	1,063	1.154 (0.926–1.438)	0.203	1.276 (1.010–1.613)	0.041	1.089 (0.873–1.358)	0.450	1.195 (0.943–1.515)	0.140
Very low	561	1.225 (0.937–1.603)	0.138	1.340 (1.002–1.792)	0.049	1.490 (1.150–1.930)	0.003	1.440 (1.083–1.913)	0.012
**Sleep quality**									
Good	1,663	Reference	–	Reference	–	Reference	–	Reference	–
Poor	1,587	1.901 (1.601–2.258)	<0.001	1.824 (1.526–2.181)	<0.001	2.687 (2.265–3.188)	<0.001	2.445 (2.043–2.927)	<0.001

UOR, unadjusted odd ratios; AOR, adjusted odd ratios. CI, confidence interval.

AOR adjusted for gender, age, marital status, empty nest status, family history of diseases, BMI categories, physical activity level, drinking status, and smoking status.

### The association of different combinations of SES and sleep quality with multimorbidity

The group with very high SES and good sleep quality was used as the reference group, after adjusting for confounders. The combinations of each type of SES and poor sleep quality and the combination of very low SES and good sleep quality were significantly associated with the prevalence of multimorbidity. Among these groups, older adults with low SES and poor sleep quality had the highest risk of the prevalence of multimorbidity (OR = 3.139, 95% CI: 2.288–4.307) (Table 4).

**Table 4:** Association of different combinations of SES and sleep quality with multimorbidity in older adults.

			**Types of chronic diseases**							
**SES**	**Sleep quality**	**N**	**0 vs. 1**				**0 vs**. ≥**2 (multimorbidity)**			
			**UOR (95%CI)**	***P***	**AOR (95%CI)**	***P***	**UOR (95%CI)**	***P***	**AOR (95%CI)**	***P***
Very high	Good	517	Reference	–	Reference	–	Reference	–	Reference	–
	Poor	356	1.932 (1.383–2.699)	<0.001	1.706 (1.204–2.418)	0.003	2.441 (1.755–3.394)	<0.001	2.031 (1.433–2.880)	<0.001
High	Good	415	0.990 (0.725–1.352)	0.949	1.001 (0.724–1.385)	0.995	0.796 (0.570–1.111)	0.181	0.829 (0.583–1.177)	0.294
	Poor	338	1.674 (1.190–2.353)	0.003	1.678 (1.175–2.396)	0.004	2.269 (1.628–3.161)	<0.001	2.098 (1.474–2.987)	<0.001
Low	Good	495	1.100 (0.818–1.479)	0.529	1.154 (0.845–1.575)	0.368	0.918 (0.671–1.256)	0.594	0.920 (0.660–1.283)	0.624
	Poor	568	2.396 (1.778–3.228)	<0.001	2.529 (1.847–3.464)	<0.001	3.109 (2.313–4.180)	<0.001	3.139 (2.288–4.307)	<0.001
Very low	Good	236	1.321 (0.903–1.933)	0.152	1.409 (0.943–2.107)	0.095	1.719 (1.188–2.487)	0.004	1.629 (1.097–2.418)	0.015
	Poor	325	2.177 (1.528–3.103)	<0.001	2.203 (1.509–3.216)	<0.001	3.318 (2.360–4.665)	<0.001	2.787 (1.926–4.034)	<0.001

UOR, unadjusted odd ratios; AOR, adjusted odd ratios. CI, confidence interval.

AOR adjusted for gender, age, marital status, empty nest status, family history of diseases, BMI categories, physical activity level, drinking status, and smoking status.

## Discussion

In this study, the prevalence of multimorbidity was 30.31% in older adults. Studies on multimorbidity have been conducted extensively in China and worldwide, but the estimate for the prevalence of multimorbidity varies widely in different studies. A cross-sectional study conducted in northeastern China showed that the prevalence of multimorbidity was 24.7% in adults, with the prevalence increasing with age, and up to 51.2% in older adults aged 60–79 years (32). In Yunnan Province, China, the prevalence of multimorbidity among rural older adults aged 60 and above was 16.1% (33). This large variation has been observed in studies from other countries and regions, for example, in South Asia, the prevalence of multimorbidity ranges from 4.5 to 83% (34). The inconsistency of the prevalence of multimorbidity may be related to the differences in the characteristics of participants and types of chronic disease in different studies. Although the prevalence of multimorbidity was inconsistent, it remained at a high level in this study. In the context of increasing aging, it is important to pay more attention to the prevalence of multimorbidity of older adults to improve their health and ensure that they enjoy their old age.

This study found that there was a negative correlation between SES and the prevalence of multimorbidity; very low SES increased the risk of the prevalence of multimorbidity in older adults. Kivimäki et al. (35) found that low SES was associated with 18 of 56 specific diseases or health conditions, including diabetes, heart failure, and obesity, suggesting that SES has an influence on the development and prevalence of multimorbidity (36). A systematic evaluation (24 articles were included) found that low education level and high deprivation were associated with an increased risk of multimorbidity (5). Older adults with lower SES in this study tend have lower educational level, poorer occupation before retirement, or lower income, and these factors are associated with multimorbidity prevalence (5, 37, 38). Kuo et al. (39) confirmed inequities in healthcare utilization among people with different occupations and income levels. Older adults with lower SES have difficulty accessing quality healthcare services, which adversely affects their health. Meanwhile, the lower SES group may adopt unhealthy lifestyles, such as smoking, risky drinking, and insufficient physical activity, which are not conducive to maintaining health (11, 40, 41). In addition, the knowledge and awareness of chronic disease prevention and control of less educated people may be even lower (42), increasing the risk of developing multimorbidity.

The older adults with poor sleep quality accounted for 48.83% in this study, and the results also found that poor sleep quality was related to the prevalence of multimorbidity in older adults. A cross-sectional study in Cypriot found that people with poor sleep quality had a higher risk of developing two, three, or more than three morbidities (43). Moreover, Sindi et al. (44) confirmed that moderate to severe sleep disturbances were associated with a higher speed of chronic disease accumulation in older adults without multimorbidity at baseline, including neuropsychiatric and musculoskeletal diseases. With the onset of aging, sleep quality of the older adults gradually decreases, which may be due to the fact that circadian oscillations become less pronounced in older adults and melatonin levels decrease, making older adults go to sleep earlier and wake up earlier than younger adults, as well as making it more difficult for them to fall asleep and stay asleep (18).

In addition to poor sleep quality due to aging, several studies have found that lower SES is associated with poorer sleep quality (24–26). Habitual attaining of adequate sleep is one of key components of a healthy lifestyle (45), and Xue et al. (23) indicated that there was a significant positive correlation between SES and health promoting lifestyle of the older adults. Older adults with lower SES may not give their lifestyles much attention, which may make it difficult for them to get a good night sleep. Older adults with lower SES may live in adverse material circumstances, such as crowded rooms, excessive noise, and bad public security, which can have direct effects on sleep quality (46). In addition, social isolation is particularly frequent in disadvantaged socioeconomic groups, and older adults with social isolation may experience sleep problems that affect sleep quality (47, 48).

The association of different combinations of SES and sleep quality with the prevalence of multimorbidity was analyzed after analyzing the association of them with the prevalence of multimorbidity separately. Older adults with very high SES and good sleep quality were used as the reference group. When the association between SES and the prevalence of multimorbidity was analyzed separately, only very low SES was significantly associated with the prevalence of multimorbidity. However, when poor sleep quality was combined, the risk of the prevalence of multimorbidity was significantly higher in every SES group. Meanwhile, very low SES was significantly associated with the prevalence of multimorbidity, regardless of whether the sleep quality was good or poor. The results suggested that it is necessary for older adults to pay more attention to their sleep status and ensure better sleep quality, in order to reduce the risk of the prevalence of multimorbidity. Meanwhile, for older adults with very low SES, more comprehensive measures should be given to protect their health, considering that the negative impact of very low SES on their health may outweigh the positive impact of good sleep quality.

## Limination

This study has several limitations to mention. First, we used a cross-sectional design for investigating the impact of SES and sleep quality on the prevalence of multimorbidity, so it is not possible to establish causal relationships. Second, the sample is not sufficiently representative. All the participants were older adults and were from the same province, which means that the results may be unique. In addition, we used the PSQI to measure sleep quality in older adults, and the results did not constitute a clinical diagnosis of poor sleep quality.

## Conclusion

This study investigated the impact of SES and sleep quality on the prevalence of multimorbidity in older adults through a survey among 3,250 older adults. The results showed that the risk of the prevalence of multimorbidity in older adults increased significantly with decreasing SES and sleep quality. The combined association of SES and sleep quality suggested that improving sleep quality is an effective health promotion modality for most older adults, while those at the bottom of the society may require more comprehensive measures to protect their health.

## Data availability statement

The raw data supporting the conclusions of this article will be made available by the authors, without undue reservation.

## Ethics statement

The studies involving human participants were reviewed and approved by Shanxi Medical University. The patients/participants provided their written informed consent to participate in this study.

## Author contributions

CZ designed and organized this survey. BX, YX, FD, SX, and JZ collected and cleaned the data. BX, YX, and FD analyzed the data and wrote the manuscript. XZ, LS, WO, and QW revised and edited the manuscript. All authors have read and approved the final manuscript.

## Funding

This work was supported by the National Natural Science Foundation of China [Grant Number: 71874104], Guangdong Basic and Applied Basic Research Foundation [Grant Number: 2022A1515011591], Key Laboratory Development Project for Philosophy and Social Sciences in Guangdong [Grant Number: G620369695], National Key R&D Program of China [Grant Number: 2020YFC2006400], and Shenzhen Social Science Planning Project [Grant Number: SZ2020B019].

## Conflict of interest

The authors declare that the research was conducted in the absence of any commercial or financial relationships that could be construed as a potential conflict of interest.

## Publisher's note

All claims expressed in this article are solely those of the authors and do not necessarily represent those of their affiliated organizations, or those of the publisher, the editors and the reviewers. Any product that may be evaluated in this article, or claim that may be made by its manufacturer, is not guaranteed or endorsed by the publisher.
